# Supplementary material for: Evaluating the quality of life among melasma patients using the MELASQoL scale: A systematic review and meta-analysis
Source: PLoS One. 2022 Jan 27;17(1):e0262833. doi: 10.1371/journal.pone.0262833 (PMC8794204; doi:10.1371/journal.pone.0262833)
Supplement: S1 Table — (DOCX) [file pone.0262833.s002.docx]

**S1 Table. Search strategy**

| **Databases** | **Search terms** | **Date** |
| --- | --- | --- |
| PubMed | (chloasma OR melasma OR melanosis) AND (quality OR questionnaire OR scale OR MELASQoL OR epidemiologic)  Search option: All fields | February 1, 2021 |
| Cochrane library | (chloasma OR melasma OR melanosis) AND (quality OR questionnaire OR scale OR MELASQoL OR epidemiologic)  Search option: All fields | February 1, 2021 |
| Web of Science | (chloasma OR melasma OR melanosis) AND (quality OR questionnaire OR scale OR MELASQoL OR epidemiologic)  Search option: All fields | February 1, 2021 |
| Embase | (chloasma OR melasma OR melanosis) AND (quality OR questionnaire OR scale OR MELASQoL OR epidemiologic)  Search option: All fields | February 1, 2021 |
