# Supplementary material for: Evaluating the quality of life among melasma patients using the MELASQoL scale: A systematic review and meta-analysis
Source: PLoS One. 2022 Jan 27;17(1):e0262833. doi: 10.1371/journal.pone.0262833 (PMC8794204; doi:10.1371/journal.pone.0262833)
Supplement: Attachment — Submitted filename: Response to Reviewers.docx [file pone.0262833.s003.docx]

**Dear academic editor and reviewers:**

**Thank you very much for giving us an opportunity to revise our manuscript. We appreciate the editor and reviewers very much for their constructive comments and suggestions on our manuscript entitled** **“Evaluating quality of life among melasma patients with the MELASQOL scale: a systematic review” (****ID: PONE-D-21-20728).**

**Those comments are very helpful for revising and improving our paper, as well as the important guiding significance to other research. We have studied the comments carefully and made corrections which we hope meet with approval. The main corrections are in the manuscript and the responds to the reviewers’ comments are as follows (the replies are highlighted in blue).**

**Replies to the reviewers’ comments:**

**Reviewer #1:**

**1. Did the authors consider doing a scoping review as a precursor to a systematic review to map the literature, identify the types of studies available.**

**Response：After reviewing relevant literatures and conducting serious discussions, we think that the available literature types of current studies are relatively clear (literatures that use MELASQOL scale to evaluate the quality of life of patients with melasma), so we do not plan to do a scope review for the time being. Thank you very much for your effective suggestions.**

**2. Inclusion and exclusion criteria lacks clarity.**

**Response：According to your suggestion, we have added inclusion and exclusion criteria, hoping to better serve the purpose of the article.**

**3.** **Lines 144-146: "Despite the mixed result between the MELASQOL and MASI scores, the articles in this review explained that the burden from melasma is obviously influenced by psychosocial distress associated with the depigmentation itself". Did the authors mean hyperpigmentation?**

**Response：We are very sorry for the wrong meaning of words in the article. Thank you for reminding us that we really mean hyperpigmentation.**

**4. Though the authors have stated that the included studies are heterogeneous under' limitations', they have not discussed the same in depth from a qualitative point of view.**

**Response: After your reminder, we realized that only part of the article discussed the results of the review from a qualitative perspective, so we repositioned the article as a systematic review.**

**Thank you again for your suggestions and opinions on our article, and we hope to get your suggestions again after revision.**

**Reviewer #2:**

**1. The title should include MELASQOL, since you have excluded other studies on QoL which did not use MELASQOL.**

**Response: According to your suggestion, we have revised the title of the article** as “**Evaluating quality of life among melasma patients with the MELASQOL scale: a systematic review”**

**2. Not familiar with the term gray literature. Please clarify.**

**Response: Grey literature generally refers to the literature not published publicly. There are a wide variety of grey literature, including non-publicly published government documents and academic dissertations; conference documents, scientific and technological reports and technical archives; unpublished manuscripts and internal publications, etc. Although some gray literature information is not mature, but the information involved is extensive, novel content, unique views, has special reference value. In our included literatures, this part of literatures was included in order not to cause defects.**

**3. There are several English language errors. Please address them carefully.**

**Response: We are really sorry for the language error. Before submitting the manuscript this time, we conducted professional language polishing service (Wiley Editing Services: CLTTX_1_5), hoping to solve this problem.**

**4. Though mentioned how many points your included studies had, it is not clear what does it mean? Were these good quality studies? What about risk of bias?**

**Response: We rediscussed and changed the literature quality evaluation tool, described the quality of articles and the risk of bias.**

**5. The data in Table 1 is not clearly visible, particularly the 1st alphabet / figure is blocked by the column line.**

**6. What statistical test was used to test the correlation between MASI and MELASQOL. Please be specific. What about the strength of association, based on the value of correlation coefficient? Only providing p-values is not enough.**

**Response: We edited Table 1, and supplement the missing statistical information.**

**7. p10, line 146 - 'depigmentation' Pls check.**

**Response: Corrected words with incorrect meanings.**

**8. Please provide a brief review on MELASQOL in the introduction part. Focus on its development and validation. How is it better than general skin instruments such as DLQI and Skindex? What domains of life does MELASQOL cover?**

**Response: In the introduction, we added a review of MELASQOL scale, supplemented its development and validation, domains covered, and reasons for its superiority over other general skin instruments.**

**9. Please provide SD and range along with mean values.**

**Response: We provide SD and range along with mean values.**

**10. The authors mention the effect of melasma on QoL as 'significant' and 'enormous' at various places. On what basis? What does a MELASQOL score of 46 mean? Has the interpretability of MELASQOL been studied?**

**Response: In the description and verification of many references, we believe that melasma has a significant impact on the quality of life, which is also the result of our review. After your reminding, we noticed that the mean score of MELASQOL is 46 is not so significant, so we deleted this part. The interpretability of MELASQOL is described in the discussion section.**

**11. You have mentioned physical health of patients was prominently affected? Which item of MELASQOL deals with this aspect? Is it the item 'less vital or productive'? If so, the score for this item is one of the lowest (Fig 2).**

**Response: In a part of the included literatures, an open-ended question-answering method was used for investigation, and it was found that many patients believed that melasma was a manifestation of health problems and would also affect their health. We explained this in the article.**

**12. The term 'personal history' is not clear. Variables such as age, gender, education etc are considered to be patient characteristics.**

**Response: We redefined “personal history” as “patient characteristics”.**

**13. Overall, results are not reported very well and appear superficial. It is not clear how many studies reported on which variable. Further, certain aspects are not very amenable to qualitative analysis (age, education etc). Variables such as age, education, course of disease, mental illness etc are just thrown in without going into any details. What do the authors mean by course of illness? Please also see my comment about reporting of p-values.**

**14. Table 3 is not clear. Headings are not proper.**

**Response: Since we could not reach a consistent conclusion from the viewpoints of the included literature, we could only state their respective viewpoints. Table 3 has been re-edited to list the specific conclusions of each study and give new explanations, hoping to clearly express our meanings.**

**15. The authors explanation regarding patients who have received treatment earlier having a higher MELASQOL score is not very clear. Patients who are more disturbed are more likely to seek treatment from more than one doctor (that is why they have received treatment earlier).**

**Response: This part is reformulation in the “results” and “discussion” section.**

**16. How does the QoL differ between countries? Between patients with diff skin types?**

**Response: We summarized and found no differences in quality of life between countries or patients with different skin types.**

**17. Was the study protocol registered at PROSPERO before the start of study?**

**Response: We're not registered on the PROSPERO**

**Once again, thank you very much for your constructive comments and suggestions which would help us both in English and in depth to improve the quality of the paper.**

**We look forward to hearing from you soon.**

**Best wishes,**

**Wenzhong Xiang**

**E-mail: xiangwenzhong@126.com**
