# Supplementary material for: Evaluating the quality of life among melasma patients using the MELASQoL scale: A systematic review and meta-analysis
Source: PLoS One. 2022 Jan 27;17(1):e0262833. doi: 10.1371/journal.pone.0262833 (PMC8794204; doi:10.1371/journal.pone.0262833)
Supplement: Attachment — Submitted filename: Response to Reviewers.docx [file pone.0262833.s004.docx]

**Dear academic editor and reviewers:**

**Thank you very much for giving us an opportunity to revise our manuscript again. We appreciate the editor and reviewers very much for their constructive comments and suggestions on our manuscript entitled** **“Evaluating quality of life among melasma patients with the MELASQoL scale: a systematic review and meta-analysis” (****ID: PONE-D-21-20728).**

**Those comments are very helpful for revising and improving our paper, as well as the important guiding significance to other research. We have studied the comments carefully and made corrections which we hope meet with approval. The main corrections are in the manuscript and the responds to the reviewers’ comments are as follows (the replies are highlighted in blue).**

**Replies to the reviewers’ comments:**

**Reviewer #1:**

**Thank you for your previous suggestions and your recognition now. We have revised the language again, hoping to solve the grammatical errors.**

**Reviewer #2:**

**1. domains of MELASQOL, Interpretability of MELASQOL, what is understood by 'course of disease'**

**In the “introduction”, we reintroduce MELASQoL, including his domains and interpretability. We corrected the semantics to change the “course of disease” to the “duration of disease”.**

**2.** **The inclusion/exclusion criteria for the purpose of a systematic review is relevant for studies only. How is it relevant for participants? How would the authors exclude participants not meeting the criteria, as information for every participant is generally not available?**

**We re - describe the inclusion and exclusion criteria, the part of “for participants”, it was the criteria that mentioned in each of the included study.**

**3. English language issues are still present. It may not be enough to be just grammatically correct, appropriate emphasis at important places should also be given. Overall, the manuscript is not easy to read.**

**We polish the article again by “Wiley Editing”, hope to make it easier to read.**

**4. The authors seem to have misinterpreted the point regarding 'physical health'.**

**We edited sections of the article, redescribing the impact of melasma on quality of life and removing references to “physical health”. We agree with you and thank you for your useful suggestion.**

**5.** **"We summarized and found no differences in quality of life between countries or patients with different skin types" Where is the supportive data in the manuscript? Table 1 shows a wide range of scores from different countries. How did you compare them?**

**We added a comparison of the quality of life of melasma patients in different countries (Table 4).**

**6. Table 1 - Why is the study by Yalamanchili included? You have not shown the MASI or MELASQOL scores from this study.**

**We explain this in Table 1, “The study did not provide the average value, but it did give specific scores.”**

**7. A suggestion to authors - Instead of presenting the results study-wise, try presenting them for each patient variable (summarizing results of different studies for each variable) - Age, disease duration, previous treatment etc. That will give a clearer picture.**

**We have accepted your suggestion, edited table 3 again, and eliminated results that exist any doubts or without p value.**

**8. Fig 2 - How many patients' data available / used for this? Is it okay to create an average score like this? If so, what about the average of total MELASQOL score?**

**Thank you very much for your reminding, which made us reconsider. After reading a large number of relevant literatures, we thought that a meta-analysis might be possible. The results and statistical methods are more accurate.**

**Once again, thank you very much for your constructive comments and suggestions which would help us to improve the quality of the paper.**

**We look forward to hearing from you soon.**

**Best wishes,**

**Wenzhong Xiang**

**E-mail: xiangwenzhong@126.com**
