# Supplementary material for: Evaluating the quality of life among melasma patients using the MELASQoL scale: A systematic review and meta-analysis
Source: PLoS One. 2022 Jan 27;17(1):e0262833. doi: 10.1371/journal.pone.0262833 (PMC8794204; doi:10.1371/journal.pone.0262833)
Supplement: Attachment — Submitted filename: Response to Reviewers.docx [file pone.0262833.s005.docx]

**Dear academic editor and reviewers:**

**Thank you very much for giving us opportunities to revise our manuscript again. We appreciate the editor and reviewers very much for their constructive comments and suggestions on our manuscript entitled** **“Evaluating the quality of life among melasma patients using the MELASQoL scale: a systematic review and meta-analysis” (****ID: PONE-D-21-20728R2).**

**We are very grateful that with your help, our review reads better overall after the second revision. We appreciate all the opportunities we've been given.**

**This time, we still tried our best to meet your requirements and modified the review to the best of our ability. We have studied the comments carefully and made corrections which we hope meet with approval. The main corrections are in the manuscript and the responds to the academic editor and reviewers’ comments are as follows (the replies are highlighted in blue).**

**Replies to the academic editor’s comments:**

1.The language still needs improvement. Please do a thorough recheck and revision for language and grammar.

Once again, we have overhauled the article and done professional language polishing (Wiley Editing Services with order number:CLTTX_1_7) to address language issues.

2.For the inclusion /exclusion criteria - you mention "For publication type: all article types (including original article, review, letter, etc.)" did you mean systematic reviews? This is not clear. Also, as I understand, you mention inclusion of an editorial.

The inclusion criteria are divided into two parts. The first is the inclusion criteria for systematic reviews. For publication type, we included all publication types, including letters, abstracts, editorials, etc. Then we further screen out eligible for meta-analysis from the included in the systematic reviews. Of the 14 studies (including nine original articles, two letters, two conference abstracts, and one editorial) that were included in the systematic review, four were also included in the meta-analysis.

**Once again, thank you very much for your constructive comments and suggestions which would help us to improve the quality of the paper.**

**We look forward to hearing from you soon.**

**Best wishes,**

**Wenzhong Xiang**

**E-mail: xiangwenzhong@126.com**
